# Clinical Presentation, Management, and Outcome in Patients With Myasthenia Gravis: A Retrospective Study From Two Tertiary Care Centers in Saudi Arabia

**DOI:** 10.7759/cureus.20765

**Published:** 2021-12-27

**Authors:** Hussein Algahtani, Bader Shirah, Ali Alshehri, Abdulaziz N Al Hassani, Hosam H Binseddeq, Rayan M Mukhtar, Bashar Saleh, Jamal A Taj

**Affiliations:** 1 Department of Medicine, Neurology Section, King Abdulaziz Medical City, Jeddah, SAU; 2 College of Medicine, King Saud Bin Abdulaziz University for Health Sciences, Jeddah, SAU; 3 Research Office, King Abdullah International Medical Research Center, Jeddah, SAU; 4 Department of Neuroscience, King Faisal Specialist Hospital and Research Centre, Jeddah, SAU; 5 Neuromuscular Integrated Practice Unit, Neuroscience Centre, King Faisal Specialist Hospital and Research Centre, Riyadh, SAU

**Keywords:** thymectomy, clinical features, disease course, clinical pattern, myasthenia gravis

## Abstract

Introduction: A limited number of research studies were published to delineate the clinical pattern of myasthenia gravis in Saudi Arabia. This paper is an attempt to describe some of the clinical aspects related to this disease in two large centers from two main cities in Saudi Arabia.

Methods: A retrospective multi-center observational study of patients diagnosed with myasthenia gravis was conducted. The study setting was King Abdulaziz Medical City in Jeddah and Riyadh, Saudi Arabia. The study period was 12 years, starting from January 2007 to May 2019.

Results: A total of 144 patients were included in this study (60 males and 84 females). The most common symptoms at diagnosis were ocular symptoms in 118 patients (81.9%), diplopia in 84 patients (58.3%), and/or blurred vision in 30 patients (20.8%). The majority had positive anti-acetylcholine receptor antibodies (72.2%). Pyridostigmine was the most prescribed medication for 136 patients (94.4%). Immunosuppressive medications were prescribed for 114 patients (79.2%). Around 40% of patients had exacerbations, and approximately 20% were admitted to the ICU. Thymectomy was performed for 97 patients (67.4%).

Conclusion: The present study indicates that the clinical presentation and management of myasthenia gravis remained the same in the last few years despite the introduction of new modalities of diagnosis such as the anti-muscle-specific kinase (anti-MuSK) and other autoantibodies tests. Furthermore, we observed that the number of exacerbations and ICU admission were high, which may indicate inadequate therapy. We are stressing the need for establishing specialized neuromuscular clinics with neurologists trained in neurophysiology to improve the diagnostic accuracy and outcomes for patients with myasthenia gravis.

## Introduction

The neuromuscular junction is a specialized prototypic synapse with a complex structural and functional organization that differs from those of the central nervous system. It is a cleft that contains a basal lamina that separates the unmyelinated motor nerve terminals from the postsynaptic membrane [1]. Myasthenia gravis is an acquired autoimmune disorder of the neuromuscular junction that results from an autoimmune attack of postsynaptic structures and is characterized by fatigable weakness of the voluntary muscles [2]. In western countries, the prevalence of myasthenia gravis is 50 to 125 persons per million with a bimodal age pattern of incidence (a peak in young women aged about 30 years and then another peak in older men aged about 60 years) [3]. Few studies were published to delineate the epidemiology, clinical profile, diagnostic tools, management, and outcome of myasthenia gravis in Saudi Arabia [4-7]. This paper is an attempt to describe some of these aspects in two large centers from two main cities in Saudi Arabia.

## Materials and methods

Study design

A retrospective multi-center observational study was conducted at King Abdulaziz Medical City in Jeddah and Riyadh, Saudi Arabia. Data collection started in May 2019 and was completed in October 2019.

Inclusion and exclusion criteria

The inclusion criteria were composed of all patients diagnosed with myasthenia gravis based on the clinical presentation and serological testing over 12 years, starting from January 2007 to May 2019. Patients with incomplete data about the diagnosis were excluded.

Data collection

Data were collected by the researchers from the electronic records and the patient’s files in the medical record department at King Abdulaziz Medical City using a data collection sheet. The data collection sheet included patient demographic profile (age, gender, nationality, etc.), clinical characteristics (symptoms, signs, etc.), diagnosis and workup (bedside tests, electrophysiological tests, serological tests, and thymus imaging), treatment (medical and surgical), and prognosis.

Statistical analysis

All numerical values obtained from each item of the data collection sheet as well as the demographic data were computed and presented by simple descriptive statistical tests (mean and standard deviation), frequency, and percentage. The IBM SPSS Statistics for Windows, Version 21.0 (Released 2012, IBM Corp, Armonk, New York) was used for data analysis.

Ethical considerations

This study was approved by the Institutional Review Board of King Abdullah International Medical Research Center with the approval number IRBC/1205/18.

## Results

**Figure 1:**
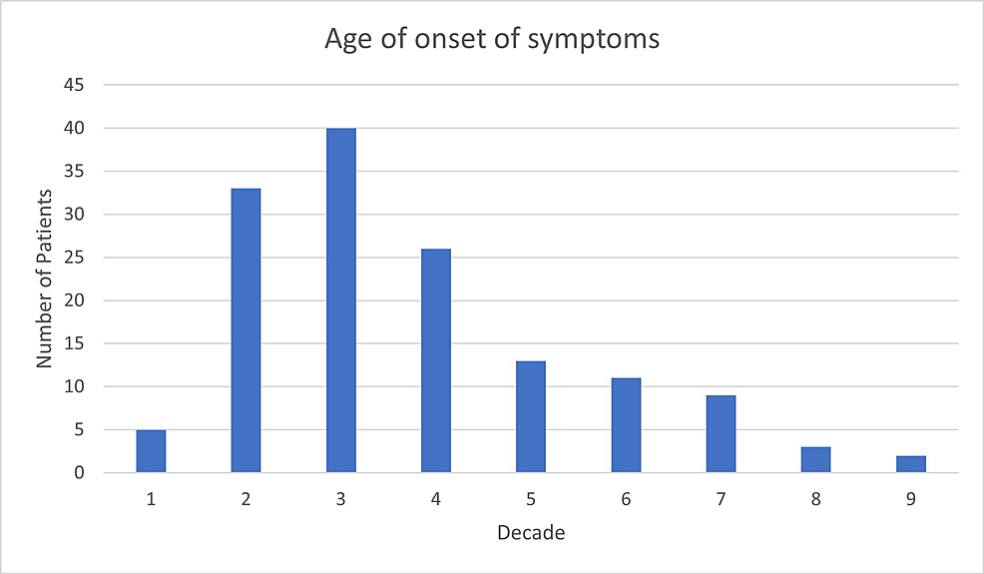
Age of onset of symptoms by decades. Each column represents a decade.

A total of 144 patients were included in this study comprising 60 males and 84 females (male to female ratio of 1:1.4). Of the patients, 79.8% were married. Age at diagnosis ranged from three years to 89 years with a mean age of 33.5±17.8 years. The onset of symptoms was seen to be maximum in the third decade, occurring in 40 patients (28%), while the age of onset at the ninth decade was only observed in two patients (1.4%) (Figure 1).

There were 17 patients (11.8%) diagnosed with other autoimmune diseases, including four patients (2.2%) with systemic lupus erythematosus (SLE), three patients (2.1%) with type 1 diabetes mellitus, and two patients (1.4%) with Graves’ disease (Table 1).

**Table 1:** Demographic details of patients with myasthenia gravis.

Variable	Patients No. (%)
Total	144 (100)
Gender	
Male	60 (41.7)
Female	84 (58.3)
Marital Status	
Single	26 (18)
Married	114 (79.8)
Divorced	1 (0.7)
Widowed	2 (1.4)
Autoimmune Diseases	
Systemic lupus erythematosus (SLE)	4 (2.8)
Type 1 diabetes	3 (2.1)
Graves’ disease	2 (1.4)
Rheumatoid arthritis	1 (0.7)
Celiac disease	1 (0.7)
Ulcerative colitis	1 (0.7)
Hashimoto thyroiditis	1 (0.7)
Vitiligo	1 (0.7)
Sjogren syndrome	1 (0.7)
Psoriasis	1 (0.7)
Autoimmune myositis	1 (0.7)

Of all 144 patients, 118 (81.9%) experienced ocular symptoms including ptosis in 89 patients (61.8%), diplopia in 84 patients (58.3%), and/or blurred vision in 30 patients (20.8%) (Table 2).

**Table 2:** Symptoms of patients with myasthenia gravis

Variable	Patients No. (%)
Total	144 (100)
Symptom	
Ocular	118 (82)
Ptosis	89 (61.8)
Diplopia	84 (58.3)
Blurred vision	30 (20.8)
Bulbar	90 (62.5)
Dysarthria	37 (25.7)
Dysphonia	25 (17.4)
Dysphagia	78 (54.2)
Nasal speech	25 (17.4)
Masticatory weakness	23 (16)
Facial weakness	29 (20.1)
Weak eyelid closure	19 (9)
Limb weakness	87 (60.4)
Upper limb weakness	79 (54.9)
Lower limb weakness	76 (52.8)
Symmetricity	73 (50.7)
Proximal weakness	86 (59.7)
Distal weakness	36 (25)
Neck weakness	21 (14.6)
Shortness of breath	39 (27.1)

The diagnosis of myasthenia gravis in these patients was established in the outpatient department for 107 patients (74.3%) while only 37 patients (25.7%) were diagnosed in the emergency department. Although 82% of patients initially developed ocular symptoms, they were all eventually diagnosed with generalized myasthenia gravis. The duration of symptoms prior to the diagnosis was one month in 12 patients (8.3%), while 18 patients (12.5%) had the symptoms for one week. Moreover, 33 patients (22.9%) had the symptoms for one to six months before the diagnosis and 18 patients (12.5%) had them for six to 12 months. Surprisingly, 13 patients (9%) reached the diagnosis in more than a year of the onset of symptoms, one of them was diagnosed after 15 years, and another one was diagnosed after 25 years. These two patients who had major delays in diagnosis were initially thought to have a functional neurological disorder. We could not document the duration of symptoms prior to diagnosis in 50 patients (34.7%).

The investigations that were done to help diagnose myasthenia gravis included bedside tests (edrophonium and ice pack), electrophysiological tests, immunological tests, and histology and tissue biopsy. Bedside edrophonium and bedside ice-pack tests were not available in this center. However, 12 patients (8.3%) have done the first and two patients (1.4%) have done the latter outside the center. Electrophysiological repetitive nerve stimulation was done in 57 patients (52.1%), but electrophysiological single-fiber electromyography was done in four patients (2.8%) only. Anti-acetylcholine receptor antibodies were positive in 104 patients (72.2%), and anti-MuSK were positive in 30 patients (20.8%). Other autoantibodies related to myasthenia gravis, such as low-density lipoprotein receptor-related protein 4 (LRP4) and anti-striational antibodies were positive in seven patients (4.9%). Of our patients, 2% were seronegative. Other tests such as antinuclear antibodies, anti-mitochondrial antibodies, anti-smooth muscle antibodies, and rheumatoid factor were done for selected patients to look for other autoimmune diseases.

**Figure 2:**
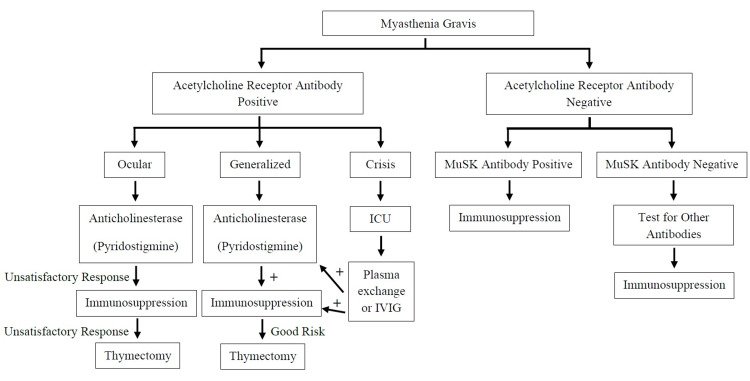
Suggested algorithm for the management of myasthenia gravis in our centers. MuSK: muscle-specific kinase; IVIG: intravenous immune globulin

Non-surgical treatment was started for all patients upon diagnosis of myasthenia gravis with pyridostigmine being the most prescribed medication for 136 patients (94.4%). Immunosuppressive medications were prescribed for 114 patients (79.2%) including glucocorticoids for 89 patients (61.8%), azathioprine for 87 patients (60.4%), mycophenolate mofetil for 14 patients (9.7%), rituximab for 12 patients (8.3%), and cyclosporine for four patients (2.8%). Only one patient (0.7%) was prescribed tacrolimus. No patients were managed with cyclophosphamide. Patients who experienced exacerbation or crisis were managed by intravenous immune globulin (IVIG), which was used for 78 patients (54.2%), and/or plasmapheresis, which was used for 41 patients (28.5%) (Figure 2).

Sixty-one patients (42.4%) had exacerbations. The number of exacerbations ranged from one to 14 times. Forty-six patients (32%) had exacerbation not exceeding five times. Twenty-nine patients (20.1%) were admitted to the ICU. The number of ICU admissions for these patients ranged from one to nine times with the majority (28 patients) being admitted two times or less. Fifteen patients (10.4%) were admitted to the ICU once, and 13 patients (9%) were admitted two times. Only one (0.7%) was admitted nine times. Forced vital capacity was measured in 54 patients (37.5%). None of the patients underwent human leukocyte antigen (HLA) typing. Three patients (2.1%) in our study died.

**Figure 3:**
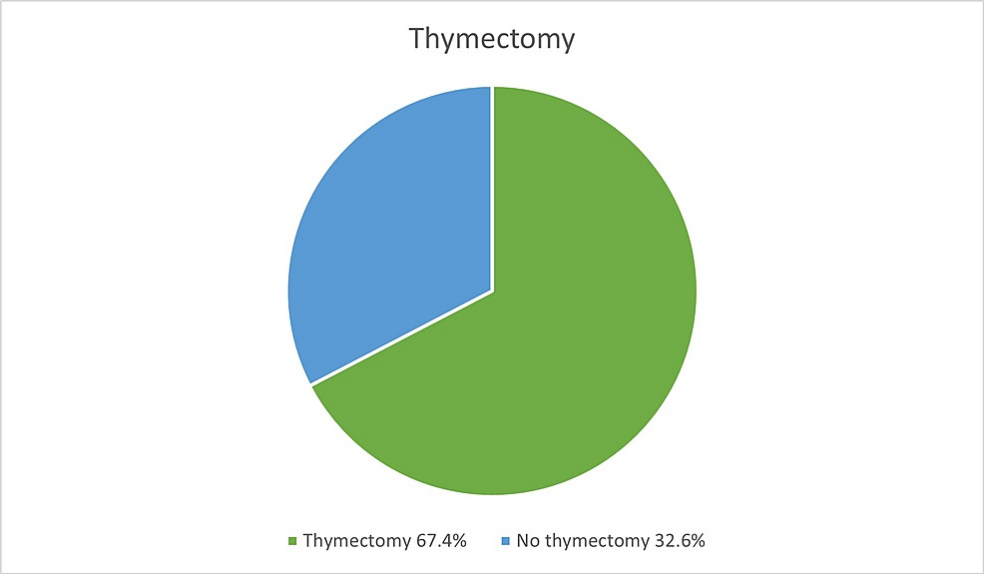
Patients who had thymectomy vs. patients who did not.

Thymectomy was done in 97 patients (67.4%); of these, 43 (44.3%) had trans-sternal thymectomy and 54 (55.7%) had videoscopic thymectomy. Thymus assessment revealed thymoma in 25 patients (17.4%), thymic hyperplasia in 31 patients (21.5%). Thymoma staging was only done in nine patients (6.3%) (Figure 3).

## Discussion

Our results showed that females are at an increased risk of developing myasthenia gravis with a male-to-female ratio of 1:1.4. This finding is consistent with a study published in 2008 by Al-Moallem et al. [4]. The peak incidence was observed in the third decade, which is consistent with the study mentioned above. Although myasthenia gravis has a bimodal distribution with a second peak occurring in the sixth or seventh decades of life, this was not observed in our study, a similar finding to Al-Moallem et al. [4].

In our study, other autoimmune disorders were observed in 10.4% of cases. The most common autoimmune disorder was SLE, thyroid gland diseases, and diabetes mellitus. This finding is less than other local figures in which 25% of patients had concomitant autoimmune diseases [4].

Almost 80% of our patients, whether males or females, were married, which is considered an interesting finding. Although the exact reasons are not well understood, we speculate that patients either get married in their second and third decade prior to being affected with the disease or it could be related to better control of the disease and its symptoms. Further studies regarding this subpopulation would be interesting.

In our study, the most common muscles involved were ocular muscles followed by bulbar/facial muscles, limb muscles, and to a lesser extent, respiratory muscles. Limb weakness involved both upper and lower limbs, both symmetric and asymmetric with more proximal weakness as compared to distal weakness. Neck muscle weakness was observed in 14.6% of cases. This is consistent with previous figures [4]. More than one-fourth of our patients complained of shortness of breath. Most of our cases were classified as grade III and grade IV according to the Myasthenia Gravis Foundation of America. Acute exacerbations were a frequent problem in our sample of patients.

The number of exacerbations was variable but the majority did not exceed five ICU admissions. About one-fifth of patients were admitted to the ICU during the course of the disease for myasthenia gravis-related symptoms.

Most of our patients were diagnosed with myasthenia gravis in the outpatient department with a lesser number of patients being diagnosed in the emergency room. The diagnosis was made within one year after the onset of symptoms in 63.8% of patients. The duration of signs and symptoms was unknown in 34.7% of cases, possibly indicating a lack of documentation. This is in contrast to a study conducted in the Philippines where the mean duration of symptoms prior to diagnosis was 21 months [8].

Bedside testing was not utilized in our centers. These include the ice pack test and edrophonium test (in collaboration with ICU). Less than 10% of cases underwent these tests, which is probably related to the tendency to diagnose patients with more specific and sophisticated tests, including antibody testing and electrophysiological modalities. Only 2% of our patients were seronegative, which is much less than the figures reported in the literature. We should be cautious with such a conclusion since we are not testing for other rare antibodies such as antibodies directed against agrin, ColQ, and Kv1.4 proteins. In addition, missing the diagnosis is another possibility, since these patients may seek other advice in other medical centers within the city. Repetitive nerve stimulation was done to almost half of our patients, unlike the single-fiber electromyography, which was only done to a very small number of patients. Our diagnostic approach is consistent with the Association of British Neurologists’ guidelines for the management of myasthenia gravis [9].

Medical treatment was initiated for all patients based on our internal algorithm after the diagnosis was established. Pyridostigmine was the most prescribed medication, followed by immunosuppressants such as glucocorticoids, azathioprine, and mycophenolate mofetil. Only a small number of cases were treated utilizing rituximab, cyclosporine, and tacrolimus. None of our patients were treated with cyclophosphamide. In acute exacerbations and refractory cases, IVIG was utilized more frequently than plasmapheresis due to availability, ease of administration (no need for central line), short duration, and the previous response of the patient. The use of such expensive and invasive modalities has been supported by a study that compared the use of IVIG and plasmapheresis, which concluded that the efficacy of both was similar in treating moderate to severe myasthenia gravis; therefore, the choice can be based on the availability of either [5].

For many years, the standard surgical approach for thymectomy has been sternotomy since it offers a good overview of the anterior mediastinum. With the advances and success of the video-assisted thoracoscopic surgery thymectomy approach to access the mediastinum, this technique became superior to the standard sternotomy technique. This technique minimizes trauma and offers a better chance of removing all thymic tissues including ectopic thymic tissues, which may be scattered in the anterior mediastinum and cervical fat. Until now, there is no convincing evidence that the response, success, and remission of myasthenic symptoms are better in sternotomy as compared to video-assisted thoracoscopic surgery. On the other hand, the transsternal approach may cause significant complications including sternal wound infection, wound dehiscence, post-operative pain, respiratory complications, and sympathetic stimulation resulting in arrhythmias and hemodynamic instabilities. Other significant complications include brachial plexus injury, pseudoarthrosis, and keloid formation with negative cosmetic effects. Another advance in the surgical management of myasthenia gravis is the development of robot-assisted thoracic surgery. This approach offers a promising and minimally invasive technique for performing thymectomy [10,11]. In our study, surgical management (thymectomy) was performed in approximately two-thirds of cases. Both techniques (transsternal and videoscopic thymectomy) were almost equally utilized in our patients. No cases were performed using the robotic technique. Although videoscopic thymectomy was not available in Jeddah, patients who preferred to undergo thymectomy utilizing this approach were sent to Riyadh to perform the procedure on the government’s expenses. Only patients who underwent thymectomy had thymus histopathology done. Thymoma and thymic hyperplasia were the reported results of histopathology in more than half of patients.

This study had some limitations related to the retrospective study design and small sample size. In spite of these limitations, these results can be used as an updated reference for the clinical pattern of myasthenia gravis and the current medical and surgical management. In addition, several findings were observed, and important conclusion remarks can be made.

## Conclusions

The clinical presentation did not change in the last few years, nor did the management. However, new modalities of diagnosing myasthenia gravis were introduced such as the anti-MuSK and other autoantibodies tests. The results of our study are an urgent call for establishing specialized neuromuscular clinics that utilize organized approaches, simple bedside tests, and more sophisticated investigation tools. More neurologists trained in neurophysiology are required to comfortably perform single-fiber electromyography. In addition, the high number of exacerbations and ICU admission could be related to the underutilization of more powerful treatment modalities such as biological therapies.
